# Integrated Biomimetic 2D-LC and Permeapad^®^ Assay for Profiling the Transdermal Diffusion of Pharmaceutical Compounds

**DOI:** 10.3390/molecules31020379

**Published:** 2026-01-21

**Authors:** Ilaria Neri, Craig Stevens, Giacomo Russo, Lucia Grumetto

**Affiliations:** 1 Department of Pharmacy, University of Naples Federico II, Via D. Montesano 49, 80131 Naples, Italy; ilaria.neri@unina.it; 2 Centre of Biomedicine and Global Health, School of Applied Sciences, Sighthill Campus, Edinburgh Napier University, 9 Sighthill Ct, Edinburgh EH11 4BN, UK; c.stevens@napier.ac.uk; 3 School of Chemistry, University College Cork, Kane Building, College Road, T12 YN60 Cork, Ireland; 4 National Institute of Biostructures and Biosystems (INBB), Consorzio Interuniversitario, Viale Medaglie d’Oro, 305, 00136 Rome, Italy

**Keywords:** skin permeation, two-dimensional liquid chromatography (2D-LC), Permeapad^®^, immobilised artificial membrane, biomimetic chromatography

## Abstract

A comprehensive two-dimensional liquid chromatography platform (LC × LC) was developed and validated for dermal permeability studies. In this implementation, the two separation dimensions were applied to mimic the layered structure of human skin: a ceramide-like stationary phase in the first dimension (^1^D) to simulate the lipid-rich epidermis, and an immobilized artificial membrane (IAM) phase in the second (^2^D) to emulate the dermis. Experimental conditions were optimised to reflect the microenvironment of the in vivo skin. For validation purposes, 43 pharmaceutical and cosmetic compounds whose transdermal permeability coefficients (log K_p_) were known from the scientific literature were selected as model solutes. A good degree of separation was achieved across the whole dataset, and affinity profiles correlated with transdermal passage properties, suggesting that retention within specific chromatographic ranges may be predictive of skin permeation. To complement this approach, mass diffusion measurements were also conducted using Permeapad^®^ 96-well plates and LC was performed on a narrow bore column in MS-friendly conditions. These log K_p_ values were compared against both in vivo and chromatographic retention data. The combined use of these techniques offers a strategic framework for profiling new chemical entities for their dermal absorption in a manner that is both ethically compliant and eco-sustainable.

## 1. Introduction

**Figure 1:**
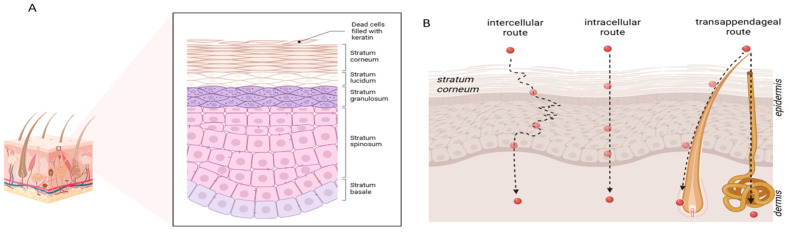
(**A**) Stratum corneum structure; (**B**) schematic representations of transdermal delivery mechanisms.

The skin, the largest organ of the human body, functions not only as a sophisticated physical barrier but also as a promising route for drug delivery. Its finely specialised three-layered structure—epidermis, dermis, and hypodermis—protects the body from toxic agents and micro-organisms, while simultaneously offering unique advantages for the systemic administration of active ingredients [1]. The uppermost layer of the epidermis comprises multiple sublayers, including the stratum corneum (SC), which consists of approximately thirty layers of dead keratinocytes embedded in a lipid-rich matrix. Owing to its composition, the SC represents the rate-limiting barrier for xenobiotic absorption [2]. Molecules may permeate the skin via either the transepidermal or transappendageal pathways. The former involves intercellular and intracellular routes—favourable to hydrophilic and hydrophobic molecules, respectively—while the latter exploits hair follicles and sweat glands, serving as the preferred pathway for large and highly lipophilic compounds (Figure 1) [3]. Once a substance permeates beyond the SC, it can readily reach the vascularised dermis, making the accurate measurement of transdermal passage rates (log K_p_) essential.

Current methodologies for assessing skin permeability rely mainly on human or animal model membranes, which often suffer from low reproducibility and limited throughput [4]. Consequently, substantial research efforts are directed toward developing reliable, sustainable, and ethical alternatives to ex vivo skin. In this context, multidimensional analytical techniques have emerged as powerful tools for emulating complex biological barriers. In particular, biomimetic bidimensional liquid chromatography (2D-LC) has recently gained increasing attention. Biomimetic chromatography uses stationary phases with biological components such as phospholipids or proteins. This allows the chromatographic system to mimic the environments where drug molecules interact, are absorbed, and are distributed [5]. Multidimensional chromatography increases separation efficiency by combining two often complementary separation mechanisms. In 2D-LC, unresolved compounds from the first dimension (^1^D) are transferred to the second (^2^D), where differences in selectivity may enable an improved separation.

Several operational modes exist in 2D-LC, including comprehensive (LC × LC) and heart-cutting (LC-LC) approaches. In LC × LC analyses, the entire ^1^D effluent is injected in small fractions in ^2^D, requiring rapid 2D separations to preserve ^1^D resolution [6]. LC-LC and multiple LC-LC, in contrast, isolate selected fractions for detailed 2D analysis, offering higher resolution, impurity detection and flexible runtime management [7,8]. A critical factor in effective ^2^D separations is orthogonality—the degree of complementarity between the two separation mechanisms. High orthogonality improves coverage of the ^2^D separation space, though excessive disparity may introduce challenges such as solvent incompatibility and viscosity mismatches [9].

Building on the potential of multidimensional and biomimetic approaches, recent studies have successfully applied LC × LC to predict human intestinal absorption by coupling biomimetic stationary phases representing distinct biological components [10]. Inspired by this strategy, the present work aims to mimic the multilayered structure of human skin through an analytical platform based on LC × LC. The system integrates an embedded polar amide column in the ^1^D, emulating the ceramide-rich SC [11], with an immobilised artificial membrane (IAM) column in the ^2^D, mimicking the phosphatidylcholine-based inner layers. To validate the platform, 43 pharmaceutical and cosmetic ingredients with known log K_p_ values [12] were analysed, and relationships between chromatographic affinity indices (log k) and skin permeability were investigated. Additionally, the 2D chromatograms were examined to identify regions containing molecules with distinct permeation properties.

In parallel, we developed an in vitro permeability assay using Permeapad^®^, a high-throughput artificial membrane that has been previously validated for assessing passive permeability in gastrointestinal, buccal, and nasal tissues [13,14]. Coupled with miniaturised high-throughput chromatography, Permeapad^®^ enabled the experimental determination of log K_p_, which was subsequently compared with literature data and with affinity chromatographic data derived from the biomimetic platform. Furthermore, in silico modelling was performed to develop predictive equations for log K_p_, to enhance the standardisation and predictive power of transdermal permeability assessments.

## 2. Results

### 2.1. Fully Biomimetic Platform Design

**Figure 2:**
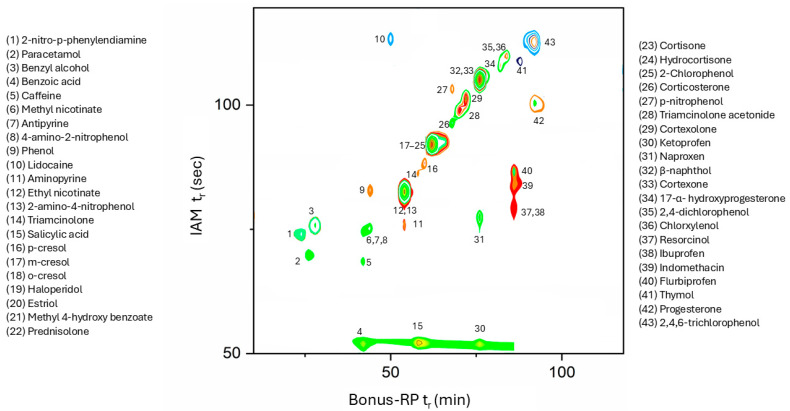
Contour plot of 43 chemicals in the dataset recorded at λ 230 nm on the Zorbax BONUS-RP × IAM.PC.DD2.

Two stationary phases, IAM and ceramide-like, embedding biological structures and mimicking the skin in a 2D-LC setting, were employed to separate 43 chemicals: (a) Zorbax Bonus-RP analytical column, which uses a unique combination of densely reacted diisopropyl-C14 groups covalently bonded through an embedded amide functionality to a silica core, and (b) IAM.PC.DD2 column based on phosphatidylcholine (PC) analogues covalently bound to a silica core end-capped with C3 and C10 anhydrides. To the best of our knowledge, the proposed experimental design has been exploited for the first time to mimic the main structures of the skin in the separation of 43 chemicals. Several experimental setups were tested before achieving a satisfactory separation across the entire dataset, as shown in Figure 2. The following adjustments in ^2^D have been explored: a decrease in modulation time (i.e., 1.50 min, 1.60 min), an increase in flow rate (i.e., 5.0 mL min^−1^), and their combinations. Adjustments in ^1^D have also been performed, including an increased flow rate (i.e., 50 μL min^−1^) and different mobile phase compositions at the start of the gradient (e.g., 100% buffer; 85% buffer). Various run times were tested, and we successfully optimised the analysis duration, reducing it by 30% compared to the initial 120 min.

Because it is well-known that when the separation mechanisms are somewhat related in the two dimensions, the best results can be obtained using a parallel gradient in 2D [15], we also explored this elution program to improve the separation of otherwise co-eluting compounds in the elution window and, in addition, to achieve chromatographic indices that could be compared using a linearly, rather than segmentally, incremented fraction of organic modifier (see Supplementary Material). Other modifications, including temperature changes, were avoided to maintain consistency with in vivo conditions and to preserve the column lifespan. Moreover, considering the significantly high pressure generated in the ^2^D analysis (≥400 bar), higher viscosity solvents, such as ethanol and methanol, were not used, even though they are occasionally employed in biomimetic studies and have a favourable carbon footprint.

### 2.2. Permeapad^®^ Passive Diffusion Evaluation

The permeability of all 43 compounds, including cationic (*n* = 1), anionic (*n* = 7), and neutral molecules, was quantified by measuring the diffused mass of each compound in the receptor chamber over time. The linear part of the cumulative curves has been used to calculate the permeability coefficient K_p_ ± SD. Table 1 reports the transdermal passage results along with the chromatographic coefficients and includes literature values for K_p_ obtained using ex vivo membranes for comparison. Under the experimental conditions applied in this study, thymol showed the lowest permeability (K_p_^Permeapad^ value −4.69), and 2-Nitro-p-phenylenediamine exhibited the most permeability (K_p_^Permeapad^ value −1.13). Furthermore, some compounds were below the limit of detection (LOD), so their permeability coefficients could not be determined. This may be due to negligible permeation under the experimental conditions.

The integration of the Permeapad^®^ barrier into a 96-well plate format provided a high-throughput platform for the parallel processing of multiple samples under fully standardised conditions. Additionally, all donor and acceptor buffers were designed to be fully MS-compatible, eliminating the need for additional sample cleanup. This combination of miniaturisation, throughput, and MS-friendly conditions makes the approach highly robust.

### 2.3. In Silico Calculations

To thoroughly investigate the experimental data obtained from the biomimetic platform, we performed in silico calculations to develop accurate predictive models.

**Figure 3:**
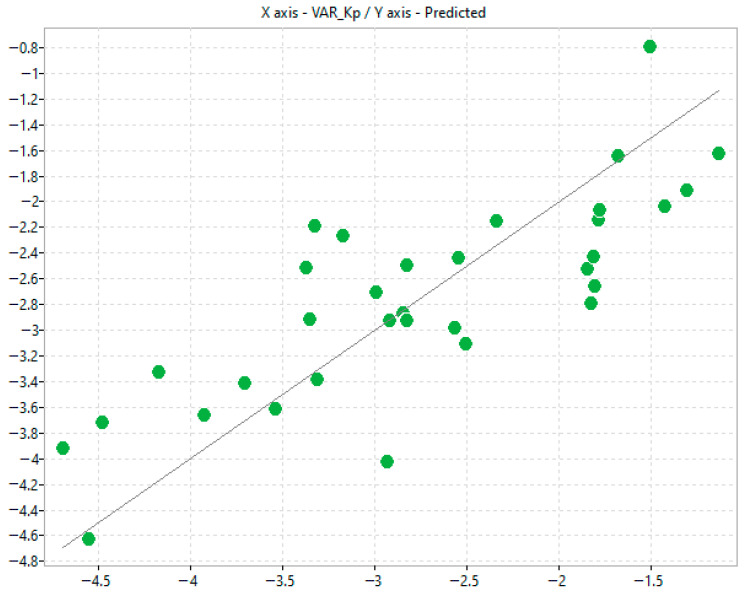
Scatter plot of predicted K_p_ and experimental Franz K_p_ values corresponding to model n.1.

An initial modelling (referred to as Model 1) was performed using the automated procedure implemented within alvaModel. The software’s built-in genetic algorithm was employed to identify the most relevant descriptors for model construction, and no manual filtering or external selection criteria were applied (Figure 3 and Table 2).

**Figure 4:**
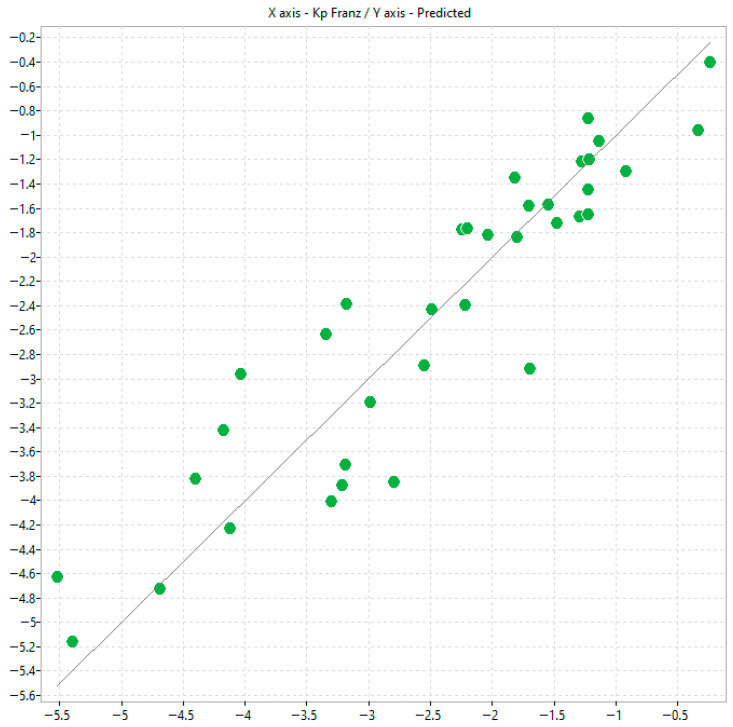
Scatter plot of predicted K_p_ and experimental Franz K_p_ values corresponding to model n.2.

By using the automatic model generation feature available in alvaModel, it was possible to leverage the option to fix specific descriptors during the feature selection process. In this study, the experimental variables log k^IAM.MG.DD2^, log k^Bonus-RP^, and log K_p_^Permeapad^ were deliberately kept constant. This approach enabled a focused evaluation of their contribution to the predictive model (Figure 4 and Table 3).

**Figure 5:**
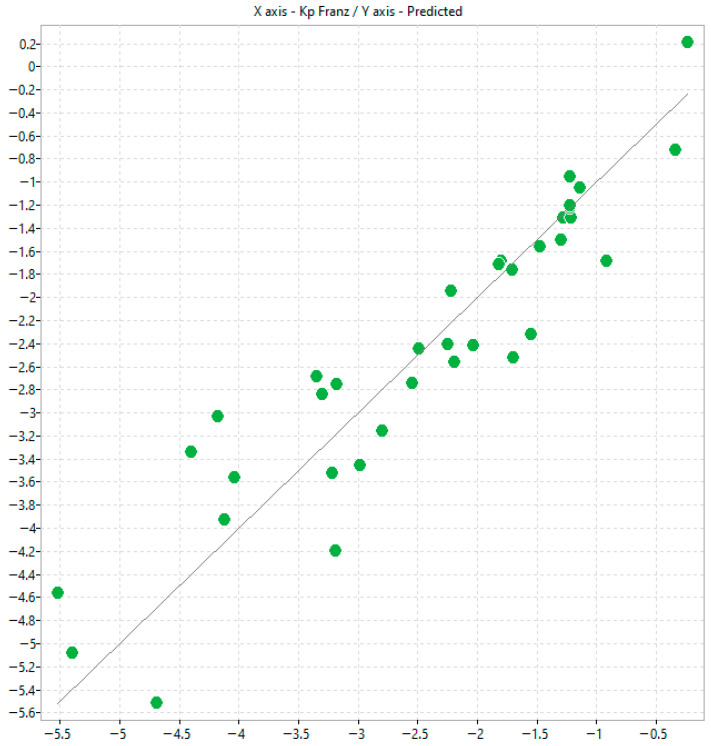
Scatter plot of predicted K_p_ and experimental Franz K_p_ values corresponding to model n.3.

Finally, to facilitate mechanistic elucidation, a carefully curated subset of descriptor categories was selected for constructing the alternative model. Specifically, 2D matrix-based descriptors, P_VSA-like descriptors, ETA indices, edge adjacency indices, and molecular properties were retained. These descriptor families were chosen for their ability to capture key structural, electronic, and physicochemical attributes of the molecules, thereby supporting a mechanistically interpretable framework for predicting permeability coefficients (Figure 5 and Table 4).

## 3. Discussion

**Figure 6:**
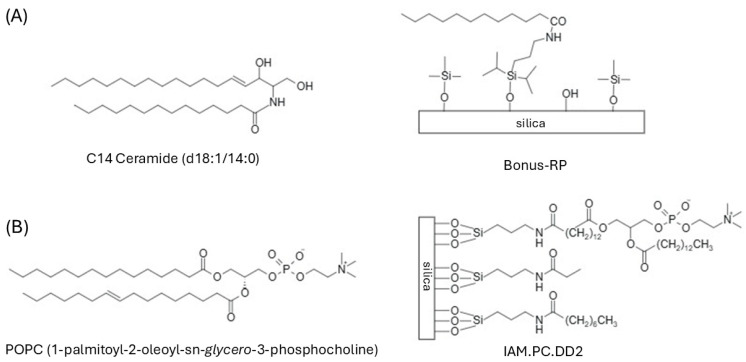
(**A**) Ceramide and ^1^D stationary phase chemistry (Zorbax Bonus RP) structures; (**B**) Phospholipid and ^2^D stationary phase chemistry (IAM.PC.DD2) structures.

The fully biomimetic platform was designed to capture complementary aspects of transdermal permeation by exploiting chromatographic affinity measurements on biologically inspired stationary phases. Figure 6 illustrates the analogy between the stationary phases and the biological structures they emulate.

Ceramides, which constitute the major lipidic component of SC, are simple sphingolipids formed by a sphingosine linked to a fatty acid via an amide bond [11], and their presence in Bonus-RP contributes to its skin-mimicking feature. Zorbax Bonus-RP was selected as a complementary and orthogonal stationary phase due to its ability to retain analytes that are poorly resolved on conventional RP materials, its compatibility with highly aqueous mobile phases, and its chemical similarity to the skin ceramides embedded in the SC [16].

IAM columns were already exploited to profile the permeation of drug-like molecules through biological membranes [17,18,19], despite being based on a monolayer of a single phospholipid. PC, as the main phospholipid component of cell membranes, closely resembles the surface of a biological cell membrane due to its combination of hydrophobic interactions, ion pairing, and hydrogen bonding interactions.

Chromatographic conditions were adjusted to reflect physiological skin environments as closely as possible: a pH 5.5 buffer in the first dimension to reproduce the acidic outer layers of the skin, and a pH 6.5 buffer in the second dimension to mirror the progressive increase in pH deeper in the epidermis [20]. However, IAM columns inherently limit method development because of the strict manufacturer guidelines regarding pH, organic modifier, and solvent ratios. For this reason, only conditions that were entirely within the recommended range were used to avoid compromising the integrity of the stationary phase.

Interestingly, compounds exhibiting favourable skin permeation (as indicated by log K_p_^Franz)^ tend to display moderate (rt > 60 s) retention in the IAM phase and medium-to-high retention (rt > 45 min) on the Bonus RP column, suggesting the presence of a potential retention cut-off across the two-dimensional platform. Notably, ketoprofen behaves as an outlier on the IAM column, despite being among the most strongly retained compounds in the Bonus RP phase. These observations support the relevance of combining the two chromatographic dimensions to capture distinct molecular interaction profiles associated with transdermal permeability.

Although these results must be seen as preliminary, the potential of this platform lies in the aspect that analytes’ permeability could be profiled in a rapid and reliable way only by looking at the position the compound’s signal occupies in the 2D-LC chromatogram, thus avoiding complex mathematical modelling. Additionally, the fact that 2D-LC can also be used for quantitative analysis paves the way for applying the same experiment for different purposes e.g., dermal permeability assessment, QC, and degradation studies.

Permeability measurements were obtained using Permeapad^®^, a biomimetic membrane consisting of a phospholipid layer (soybean phosphatidylcholine S-100) enclosed between cellulose sheets to prevent lipid leakage [21]. Previous studies have shown a strong correlation between the permeability of Permeapad^®^ and results from Caco-2 or PAMPA assays [14,22], supporting its suitability for modelling passive diffusion. Although Magnano et al. 2022 [23] pointed out certain limitations of the classical Permeapad^®^ membrane in fully mimicking the skin barrier, no more accurate alternatives—such as the ceramide and cholesterol-enriched version— were available at the time the experiments were conducted. It is nevertheless important to note that phosphatidylcholine, which constitutes the Permeapad^®^ membrane, is itself an essential component of the lipid matrix, providing a physiologically relevant environment that supports its use as a surrogate skin membrane.

Analysing the chromatographic data, a clear relationship was observed between log P and log k on Bonus-RP, indicating that analytes were separated according to their lipophilicity. However, plotting log k^Bonus-RP^ against the permeability coefficients obtained through Permeapad^®^ revealed no discernible correlation. Given the complexity of the skin and the variety of potential permeation routes, including the aqueous pore pathway [24], it is unsurprising that *n*-octanol/water lipophilicity alone cannot explain transdermal passage [25].

The in silico modelling provided some usefulness in estimating permeability coefficients using both chromatographic and mass diffusion indices. The selected physico-chemical descriptors are often complex and represent a blend of topological, electrostatic and other molecular property parameters, making mechanism elucidation challenging. However, some useful information can still be drawn. For instance, in model 3, *n*-octanol/water lipophilicity, i.e., ALOGP2, relates positively with transdermal passage consistently implying that the more lipophilic the solute, the greater the transdermal passage observed. In addition, Chi1_AEA (dm) is a connectivity-like index which depends on complex molecular attributes including, but not limited to, overall molecular dipole moment, dipole density distribution and charge separation efficiency. This descriptor relates negatively to transdermal passage suggesting that ample clusters of polar bonds or electronic heterogeneity hinder transdermal passage.

Scatter plots correlating log k^IAM.MG.DD2^ with permeability coefficients (K_p_) obtained using Permeapad^®^ similarly showed only a weak relationship. The trend was even poorer for K_p_ values derived from Franz diffusion cell (FDC) studies [12], which are inherently affected by biological variability in excised skin tissues. These results support the hypothesis that, although the polar headgroups of IAM phases accurately mimic natural biomembranes, the permeation process is governed by a more intricate interplay of interactions than cannot be recapitulated by a single phospholipid monolayer. IAM lacks the multilayered, ceramide-rich organisation characteristic of skin, while Permeapad^®^ represents a bilayer but does not replicate the lipid heterogeneity of the stratum corneum. Moreover, IAM may insufficiently capture phospho-hydrophobic interactions in ionisable molecules.

**Figure 7:**
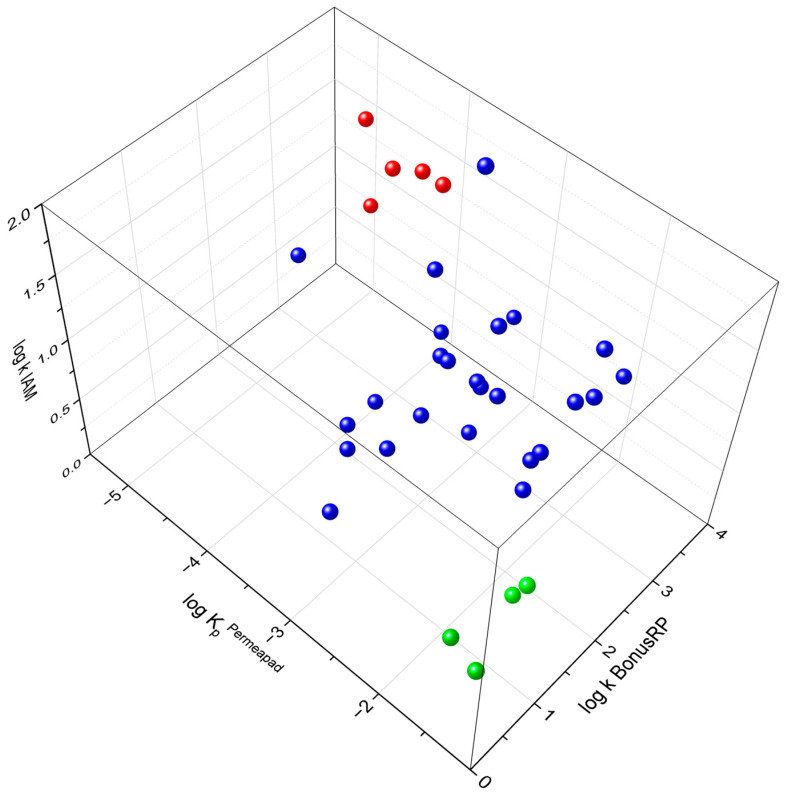
Three-dimensional plot of all experimental parameters. Compounds with a low rate of transdermal passage are shown in red, and compounds with a high rate of transdermal passage are shown in green. The compounds in blue showed an intermediate rate of transdermal passage.

Despite the absence of linear correlations, when the three experimental parameters were plotted together in a three-dimensional space (Figure 7), well-defined clusters emerged. These regions group compounds that display similar affinity patterns and comparable permeability. This multidimensional distribution demonstrates that the combined interpretation of affinity and permeability values captures trends that are not visible through pairwise comparisons alone. Such integration underscores the importance of intersecting multiple dimensions to reconstruct the multifactorial nature of skin permeation.

In this context, the platform provides added value by enabling qualitative classification of compounds based on their combined chromatographic and permeability behaviour. Overall, our findings demonstrate that while chromatographic and permeation assays independently offer only partial representations of the skin barrier, their integration, supported by modelling, provides a more holistic and fully integrated perspective. Compared with conventional dermal permeability assessment methods, which are often low-throughput, material-intensive, and dependent on complex biological models, the proposed workflow is designed as a high-throughput, microscale screening strategy suitable for early-stage compound prioritisation. In particular, the LC × LC platform, combining two stationary phases with different selectivity, enhances peak capacity and orthogonality, resulting in improved chromatographic resolution and reduced matrix effects. This enables more sensitive detection of trace components and supports a rapid, information-rich evaluation of compound–barrier interactions, thereby strengthening the platform’s potential as a screening and decision-support tool for the early evaluation of candidates for transdermal administration.

## 4. Materials and Methods

### 4.1. Chemicals and Reagents

Standard substances (quality ≥ 98%) were obtained from Fisher Scientific (Renfrew, UK), Merck KGaA (Darmstadt, Germany), SLS (Nottingham, UK) and Cayman Chemical Company (Ann Arbor, MI, USA) as listed in Table 5. Milli-Q Water (18.2 MΩ cm^−1^) was obtained in house via a water purification system (SUEZ Water Technologies and Solutions, Paris, France). HPLC-grade acetonitrile and methanol were purchased from Rathburn (Walkerburn, UK). Ammonium acetate (≥97%) for buffer preparation and phosphate buffer saline tablets were bought from Alfa Aesar (Haverhill, MA, USA) and from Sigma-Aldrich (St. Louis, MO, USA), respectively. The pH of the solutions was measured using a Mettler Toledo pH meter (Peterborough, UK) and filtered through Cole-Parmer 0.45 μm nylon membranes before use. Syringe filters with a 0.45 μm PTFE membrane from VWR International (Radnor, PA, USA) were employed to purify HPLC samples before analysis. In Table 5, all the chemicals analysed, along with their physical chemical parameters, are listed.

### 4.2. 2D LC Platform Design

The ^1^D column was a Zorbax Bonus RP (50 mm × 2.1 mm, 1.8 μm) from Agilent Technologies (Santa Clara, CA, USA), while the ^2^D column was an IAM.PC.DD2 (150 mm × 4.6 mm, 10 μm) from Regis Technologies (Morton Grove, IL, USA), placed in a thermostat column compartment. The 2D LC instrument was from Agilent Technologies (Santa Clara, CA, USA). Analysis in both dimensions was performed using a 1260 Infinity II pump coupled with a 1290 Infinity Diode Array Detector. First and second dimensions were interfaced via a 2-position/8-port switching valve equipped with an 80 μL loop. The composition of mobile phases was 10 mM ammonium acetate buffer (A) and acetonitrile (B) in both dimensions, although the pH of the ^1^D buffer was 5.5 and the pH of the ^2^D buffer was 6.5. The ^1^D separation was carried out at a 40 μL min^−1^ flow rate under a controlled temperature (30 °C) by using a linear gradient elution program set as follows: 0 min, 10% B1; 90 min, 100% B1. The ^2^D separation was performed at 4.0 mL min^−1^ at 35 °C, with a modulation time of 2.0 min, using a full gradient set as follows: 0 min, 0% B2; 1.80 min, 100% B2; 1.81 min, 0% B2; 2 min, 0% B2. Stock solutions at a concentration of 2.00 mg mL^−1^ of all compounds were prepared in methanol and stored at 4 °C. Daily working solutions were prepared at a concentration of 30 μg mL^−1^ for all analytes, using the mobile phase at the start of the gradient elution programme. Raw data registered through ChemStation^®^ C.01.07 software were converted into a data matrix using GC image 2024 software (GCimage, Lincoln, NE, USA) and then contour plots were created using Origin2024b (OriginLab Corporation, Northampton, MA, USA). The chromatographic retention index of the analytes was calculated as follows:

𝑘 = 𝑡_𝑟_ − 𝑡_0_/𝑡_0_ (1)

where *t*_*r*_ is the retention time of the analyte, *t*_0_ is the retention time of not retained substance (acetone). 

### 4.3. Permeapad^®^ Experiments

Two Permeapad^®^Plate InnoMe GmbH (Espelkamp, Germany) samples from the same batch were employed to measure the permeability coefficient of the compounds in the dataset. The 96-well plates were configured in a bottom-to-top orientation; specifically, the insert plate served as the receiving chamber and the bottom plate served as the donor chamber. The wells of the screen plate have a tilted bottom, avoiding air bubbles and increasing the area available for the permeation. Stock solutions of 100 mM or 10 mM were prepared in methanol, based on the solubility of each solute. For the permeation experiment, 400 μL of six different stock mixtures diluted in PBS (pH 5.5) were used as donor solutions, while 200 μL of PBS (pH 7.4) was placed in the receiving chamber to mimic blood. Sampling from the receiving compartments was performed in triplicate every thirty minutes for five hours. To reduce the thickness of the unstirred water layer, the plate was placed on a peQlab orbital shaker (Fareham, UK) at room temperature (20 ± 2 °C). As baseline separation was achieved for each of the designated compound mixtures, acceptor chamber samples were simultaneously quantified through HPLC analysis. In Table 6, the mixture compositions are listed as well as the donor concentration.

### 4.4. Quantitative Analysis

Standard calibration samples (ranging from 3.125 to 100.000 μg mL^−1^) along with Permeapad^®^ samples were analysed using a Waters e2695 quaternary pump coupled with a 2849 UV/Vis detector (Waters Corporation, Milford, MA, USA) set up at 230 nm. A narrow bore, core shell stationary phase RP-HPLC InfinityLab Poroshell 120 Bonus-RP (50 mm × 3.0 mm, 2.7 μm) from Agilent Technologies (Santa Clara, CA, USA) was employed, at a flow rate of 0.4 mL min^−1^ and a column oven temperature of 30 °C. A linear gradient elution program starting from 0% B to 100% B in 15 min was employed. The total run was 24 min long, including column equilibration before the next injection. Mobile phase A was 10 mM ammonium acetate and B LC MS grade acetonitrile. Version 3.7.0 Empower^®^ software was used for quantitative analysis.

### 4.5. Calculation of Skin Permeability Coefficient

The cumulative amount permeated (Q in μg) across the PermeaPad^®^ barrier per area (A, in cm^2^) was plotted against time (t in s). The linear part of the resulting graph corresponds to steady-state flux (J) [14]:

J_(flux)_ = Q/A × t (2)

To calculate K_p_ (in cm s^−1^), the steady-state flux is normalised by the donor start concentration (C_0_):

K_p_ = J/C_0_ (3)

Each compound K_p_ value is expressed as the logarithm of the mean of the individual replicates. Standard deviation values were in the range of 0.01–0.40.

### 4.6. In Silico Calculation

Lipophilicity values (log P, log D^5.5^ and D^6.5^) and the dissociation constant were calculated using Marvin Sketch 17.1.23.0 software on a personal computer. 

Alvascience, alvaDesc (software for molecular descriptors calculation) version 3.0.8, 2025, (https://www.alvascience.com, accessed on 28 November 2025) is a comprehensive software platform used for the calculation of molecular descriptors. It supports the computation of over 5000 descriptors spanning constitutional, topological, geometrical, electronic, and hybrid categories.

Alvascience, alvaModel version 2.0.18, 2025 (software to model QSAR data) was employed (https://www.alvascience.com, accessed on 28 November 2025) to construct models that predict permeability coefficients. Software was installed on a Windows 11 laptop.

The model was trained using experimental data from Franz diffusion cell assays. The automatic model generation feature available within the modelling software was chosen. AlvaModel utilises a genetic algorithm-based feature selection process to identify the most relevant descriptors for model development. Upon activation, the algorithm iteratively evaluated combinations of descriptors to optimise model performance. The process was configured to terminate when the maximum number of iterations was reached. This automated approach ensured the efficient selection of input variables and facilitated the generation of regression models tailored to the specified target.

Feature reduction was applied as follows: (a) constant value filter: descriptors with more than 95% constant values were removed; (b) variance filter: descriptors with a standard deviation lower than 0.0001 were excluded; (c) correlation filter: descriptors with an absolute pairwise correlation ≥0.95 were eliminated.

Results are expressed as r^2^ = determination coefficient RMSE = root mean square error.

## 5. Conclusions

Although these results should be regarded as preliminary, as further validation and adjustments are needed, this 2D-LC platform and the cell-free permeation models, due to their cost and time efficiency, may represent useful screening tools in the early development phases of drug discovery, as well as for newly synthesised compounds when many ingredients require characterisation. Furthermore, combining the platform and Permeapad^®^ with computer-based physicochemical data can serve as a strategic asset for assessing skin permeability in a reproducible, high-throughput, environmentally friendly, and animal-free manner. This approach is both ethical and sustainable. To further refine existing models, a holistic and collaborative approach between researchers in biology, pharmacology, and bioengineering is needed.

## Figures and Tables

**Table 1:** Logarithms of chromatographic retention factors determined via bidimensional chromatography (log k^BonusRP^ and log k^IAM.MG.DD2^), along with logarithms of permeability coefficients determined on a new membrane (K_p_^Permeapad^). K_p_^Franz^ from Grooten et al., 2022 [12] measured on animal membranes are reported. <LOQ K_p_ for these chemicals was not determined. They are listed in ascending order of permeation. Log k values were determined using a full gradient for the 2D separation.

Substances	log k^Bonus RP^	log k^IAM.MG.DD2^	log K_p_^Permeapad^ (cm/h)	log K_p_^Franz^ (cm/h)
Thymol	3.40	1.73	−4.69	−1.28
Indomethacin	3.30	1.19	−4.55	−1.30
2-Chlorophenol	2.10	1.30	−4.48	−1.48
17α-Hydroxyprogesterone	3.10	1.70	−4.17	−3.22
2,4-Dichlorophenol	3.20	1.75	−3.92	−1.22
Chloroxylenol	3.20	1.75	−3.71	−1.23
2,4,6-Trichlorophenol	3.60	1.81	−3.54	−1.23
Haloperidol	2.10	1.30	−3.49	−4.04
Naproxene	2.80	0.95	−3.37	−1.14
Cortexolone	2.60	1.53	−3.35	−4.12
4-Amino-2-nitrophenol	1.30	0.89	−3.32	−2.55
Aminopyrine	1.70	0.90	−3.31	−2.99
Methyl nicotinate	1.10	0.87	−3.17	−2.49
Ketoprofen	2.80	0.29	−2.99	−1.23
Ibuprofen	3.30	1.08	−2.93	−0.24
Methyl-4-hydroxybenzoate	2.10	1.30	−2.92	−2.04
p-Cresol	2.00	1.19	−2.85	−0.92
Benzyl alcohol	0.40	0.90	−2.83	−2.22
2-Amino-4-nitrophenol	1.70	1.06	−2.83	−3.18
Phenol	1.20	1.03	−2.82	−1.71
p-Nitrophenol	2.40	1.58	−2.57	−2.25
Hydrocortisone	2.10	1.30	−2.54	−5.52
o-Cresol	2.10	1.28	−2.51	−1.80
Estriol	2.10	1.30	−2.34	−4.40
Triamcinolone	1.90	1.16	−1.85	−5.40
m-Cresol	2.10	1.30	−1.82	−1.82
Flurbiprofen	3.30	1.19	−1.82	−0.34
β-Naphthol	2.82	1.62	−1.81	−1.55
Corticosterone	2.40	1.42	−1.78	−3.19
Ethyl nicotinate	1.70	1.06	−1.78	−2.20
Triamcinolone acetonide	2.50	1.47	−1.68	−4.69
Paracetamol	0.30	0.74	−1.51	−3.35
Lidocaine	1.50	1.84	−1.35	−1.70
Caffeine	1.10	0.70	−1.43	−2.80
Antipyrine	1.10	0.87	−1.31	−4.18
Benzoic acid	1.10	0.30	−1.13	−1.52
2-Nitro-p-phenylenediamine	0.10	0.84	−1.13	−3.30
Prednisolone	2.10	1.30	<LOQ	−4.35
Progesterone	3.60	1.50	<LOQ	−2.82
Salicylic acid	1.90	0.30	<LOQ	−2.20
Resorcinol	3.30	1.08	<LOQ	−3.62
Cortexone	3.00	1.66	<LOQ	−3.35
Cortisone	2.10	1.30	<LOQ	−5.00

**Table 2:** Model n.1 information and explanation of AlvaDesc descriptors.

Model 1		R^2^	RMSE
0.877 − 0.679 × Ho_X + 0.771 × VE3sign_Dz (p) − 3.730 × MATS3e + 0.497 × SM02_AEA (ri) − 0.494 × log k^Bonus-RP^		0.641	0.576
Name	Description	Category	
Ho_X	Hosoya-like index (log function) from chi matrix	2D matrix-based descriptors	
VE3sign_Dz (p)	Logarithmic sum of coefficients of the last eigenvector from Barysz matrix weighted by polarisability	2D matrix-based descriptors	
MATS3e	Moran autocorrelation of lag 3 weighted by Sanderson electronegativity	2D autocorrelations	
SM02_AEA (ri)	Spectral moment of order 2 from augmented edge adjacency matrix weighted by resonance integral	Edge adjacency indices	

**Table 3:** Model n.2 information and explanation of AlvaDesc descriptors.

Model 2		R^2^	RMSE
8.935 + 0.264 × Eig05_EA (bo) − 3.659 × Eig01_AEA (ri) + 0.431 × SpDiam_RG − 0.033 × K_p_^Permeapad^ + 0.910 × log k^Bonus-RP^ − 0.523 × log k^IAM.MG.DD2^		0.848	0.524
Name	Description	Category	
Eig05_EA (bo)	Eigenvalue n. 5 from edge adjacency matrix weighted by bond order	Edge adjacency indices	
Eig01_AEA (ri)	Eigenvalue n. 1 from augmented edge adjacency matrix weighted by resonance integral	Edge adjacency indices	
SpDiam_RG	Spectral diameter from reciprocal squared geometrical matrix	3D matrix-based descriptors	

**Table 4:** Model n.3 information and explanation of AlvaDesc descriptors.

Model 3		R^2^	RMSE
−0.687 + 2.078 × P_VSA_ppp_N − 0.075 × Eta_betaP − 0.266 × Chi1_AEA (dm) + 0.114 × ALOGP2 − 0.021 × K_p_^Permeapad^		0.854	0.513
Name	Description	Category	
P_VSA_ppp	P_VSA-like on potential pharmacophore points, N–negative	P_VSA-like descriptors	
Eta_betaP	Eta pi and lone pair VEM count	ETA indices	
Chi1_AEA (dm)	Connectivity-like index of order 1 from augmented edge adjacency matrix weighted by dipole moment	Edge adjacency indices	
ALOGP2	Squared Ghose–Crippen octanol–water partition coeff. (logP^2^)	Molecular properties	

**Table 5:** Properties of chemicals under analysis. pKa and log P were obtained from Drugbank, and log D was calculated using Marvin Sketch (ChemAxon Marvin Suite 17.1).

Chemicals	CAS No.	MW (g mol^−1^)	pKa	log P	log D^pH5.5^	log D^pH6.5^	Supplier
2-Nitro-phenylenediamine	5307-14-2	153.14	6.20	0.53	1.06	1.08	Fisher
17α-Hydroxyprogesterone	68-96-2	330.46	12.70	3.40	3.61	3.61	SLS
2,4,6-Trichlorophenol	88-06-2	197.40	6.23	3.69	3.27	3.01	Fisher
2,4-Dichlorophenol	120-83-2	163.00	7.90	3.06	2.80	2.78	Fisher
2-Amino-4-nitrophenol	99-57-0	154.12	7.60	1.26	0.93	0.91	Fisher
2-Chlorophenol	95-57-8	128.56	8.56	2.15	2.28	2.28	Merck
4-Amino-2-nitrophenol	119-34-6	154.12	7.81	0.96	0.92	0.90	Fisher
Aminopyrine	58-15-1	231.29	5.00	1.00	1.59	1.60	Fisher
Antipyrine	60-80-0	188.23	1.40	0.38	1.61	1.61	SLS
Benzoic acid	65-85-0	122.12	4.19	1.87	0.12	−0.84	Merck
Benzyl alcohol	100-51-6	108.14	15.40	1.34	1.21	1.21	Fisher
Caffeine	58-08-2	194.19	14.00	−0.07	−0.79	−0.79	SLS
Chloroxylenol	88-04-0	156.61	9.70	3.27	3.21	3.21	Fisher
Cortexolone	152-58-9	346.46	13.90	2.58	2.75	2.75	SLS
Cortexone	64-85-7	163.00	12.70	3.40	3.77	3.77	SLS
Corticosterone	50-22-6	346.46	13.86	2.02	2.48	2.48	SLS
Cortisone	53-06-5	360.44	12.58	1.47	2.14	2.14	Merck
Estriol	50-27-1	288.40	10.33	2.67	2.64	2.64	Fisher
Ethyl nicotinate	614-18-6	151.16	3.35	1.26	1.22	1.22	Fisher
Flurbiprofen	5104-49-4	224.26	4.03	4.16	3.54	2.67	Fisher
Haloperidol	52-86-8	375.86	8.66	3.70	0.47	1.43	SLS
Hydrocortisone	50-23-7	362.50	15.59	1.61	1.47	1.47	Merck
Ibuprofen	15687-27-1	206.28	5.20	3.97	3.09	2.18	Merck
Indomethacin	53-86-1	357.79	4.50	4.27	2.16	1.20	Cayman
Ketoprofen	22071-15-4	254.28	4.45	3.12	1.95	0.98	Merck
Lidocaine	137-58-6	234.34	7.90	2.44	0.61	1.58	Merck
m-Cresol	108-39-4	108.14	10.10	1.96	2.23	2.23	Fisher
Methyl nicotinate	93-60-7	137.14	3.24	0.83	0.88	0.88	Fisher
Methyl-4-hydroxybenzoate	99-76-3	152.15	8.50	1.96	1.91	1.91	Merck
Naproxene	22204-53-1	230.26	3.18	3.18	1.71	0.74	Merck
o-Cresol	95-48-7	108.14	10.29	1.95	2.23	2.23	Fisher
Paracetamol	103-90-2	151.16	9.46	0.46	1.09	1.09	SLS
p-Cresol	106-44-5	108.14	10.30	1.94	2.23	2.23	Fisher
Phenol	108-95-2	94.11	9.99	1.46	1.76	1.76	SLS
p-Nitrophenol	100-02-7	139.11	7.15	1.91	1.70	1.61	Fisher
Prednisolone	50-24-8	360.44	12.59	1.62	1.21	1.21	Fisher
Progesterone	57-83-0	314.46	18.92	3.87	4.63	4.63	Fisher
Resorcinol	108-46-3	110.11	9.26	1.37	1.48	1.48	SLS
Salicylic acid	69-72-7	138.12	2.97	2.26	−0.74	−1.47	SLS
Thymol	89-83-8	150.22	10.59	3.30	3.42	3.42	Merck
Triamcinolone	124-94-7	394.40	11.75	1.16	−0.60	−0.60	Fisher
Triamcinolone acetonide	76-25-5	434.50	13.40	1.94	1.92	1.92	SLS
β-Naphthol	135-19-3	144.17	9.50	2.70	2.76	2.76	Merck

**Table 6:** Permeapad^®^ mixtures with concentration values in the donor chamber expressed in mM for each substance.

Mix Number	Mix Components	Concentration in Donor Chamber (mM)
1	2-Nitro-p-phenylenediamine	1.0
	Antipyrine	1.0
	Ethyl nicotinate	1.0
	m-Cresol	1.0
	Prednisolone	1.0
	Triamcinolone acetonide	0.1
2	Benzyl alcohol	1.0
	Phenol	1.0
	2-Amino-4-nitrophenol	1.0
	p-Cresol	1.0
	Methyl-4-hydroxybenzoate	1.0
	Ketoprofen	1.0
	17α-hydroxyprogesterone	0.1
3	Paracetamol	1.0
	Caffeine	1.0
	Benzoic acid	1.0
	o-Cresol	1.0
	Haloperidol	0.1
	Corticosterone	0.1
	β-Naphthol	1.0
	Flurbiprofen	1.0
	Progesterone	0.1
4	4-Amino2-nitrophenol	1.0
	Lidocaine	1.0
	Triamcinolone	0.1
	Salicylic acid	1.0
	Estriol	0.1
	Resorcinol	1.0
	Chlorxylenol	1.0
	Ibuprofen	1.0
5	Methyl nicotinate	1.0
	Aminopyrine	1.0
	2-Chlorophenol	1.0
	Hydrocortisone	0.1
	Cortexolone	0.1
	Cortexone	0.1
	Indomethacin	0.1
6	Cortisone	0.1
	p-Nitrophenol	1.0
	Naproxene	1.0
	2,4-Dichlorophenol	1.0
	Thymol	1.0
	2,4,6-Trichlorophenol	1.0

## Data Availability

Data will be made available on request.

## Supplementary Materials

The following supporting information can be downloaded at: https://www.mdpi.com/article/10.3390/molecules31020379/s1, Figure S1: Contour plot recorded at λ 230 nm on Zorbax BONUS-RP × IAM.PC.DD2, using parallell gradient elution, of 43 chemicals in the dataset; Table S1: Logarithms of chromatographic retention factors (log k ^BonusRP^ and log k ^IAM.MG.DD2^) determined using a parallel gradient.

## Abbreviations

The following abbreviations are used in this manuscript:

^1^D	first dimension
^2^D	second dimension
2D-LC	Two-dimensional liquid chromatography
2D	two-dimensional
IAM	Immobilised artificial membrane
LC × LC	comprehensive two-dimensional liquid chromatography platform
LOD	Limit of detection
LOQ	Limit of quantification
PC	phosphatidylcholine
SC	Stratum corneum
