# Supplementary material for: Integrated Biomimetic 2D-LC and Permeapad® Assay for Profiling the Transdermal Diffusion of Pharmaceutical Compounds
Source: Molecules. 2026 Jan 21;31(2):379. doi: 10.3390/molecules31020379 (PMC12843750; doi:10.3390/molecules31020379)
Supplement: Supplementary file 1 [file molecules-31-00379-s001.zip › molecules-4042909-supplementary.pdf]

Supporting Information

Integrated Biomimetic 2D-LC and Permeapad® Assay for Profiling the Transdermal Diffusion of Pharmaceutical Compounds

Ilaria Neri [1,2,3], Craig Stevens [2], Giacomo Russo [2,*] and Lucia Grumetto [1,4,*]

1 Department of Pharmacy, University of Naples Federico II, Via D. Montesano 49, I-80131 Naples, Italy; ilaria.neri@unina.it

2 Centre of Biomedicine and Global Health, School of Applied Sciences, Sighthill Campus, Edinburgh Napier University, 9 Sighthill Ct, Edinburgh EH11 4BN, UK; c.stevens@napier.ac.uk

3 School of Chemistry, University College Cork, Kane Building, College Road, T12 YN60 Cork, Ireland

4 National Institute of Biostructures and Biosystems (INBB), Consorzio Interuniversitario, Viale Medaglie d'Oro, 305, I-00136 Rome, Italy

* Correspondence: g.russo@napier.ac.uk (G.R.); grumetto@unina.it (L.G.)

Supplementary materials

The ^{1}D column was a Zorbax Bonus RP (50 mm×2.1mm, 1.8 μm) from Agilent Technologies (Santa Clara, California, USA), while the ^{2}D column was a IAM.PC.DD2 (150 mm×4.6 mm, 10 μm) from Regis Technologies (Morton Grove, Illinois, USA), placed in a thermostat column compartment. The 2D LC instrument was from Agilent Technologies (Santa Clara, CA, USA). Analysis in both dimensions were performed with 1260 Infinity II pump coupled with 1290 Infinity Diode Array Detector. First and second dimension were interfaced *via* a 2-position/8-port switching valve equipped with 80 μL loop. The mobile phases composition was 10 mM ammonium acetate buffer (A): acetonitrile (B1 and B2) in both dimensions, even if the ^{1}D pH buffer was 5.5 (A1) and ^{2}D pH buffer was 6.5 (A2). The ^{1}D separation was carried out at a 40 μL min^{-1} flow rate and under controlled temperature (30°C) by using a linear gradient elution program set as follows: 0.0 min: 10% B1; 85.0 min: 50% B1; 90.0 min: 100% B1; 90.1 min: 10%B1; 100.0 min: 10%B1.

The ^{2}D separation was performed at 4.0 mL min^{-1} at 37°C, setting up the same gradient program as in ^{1}D. 0.0 min 0% B2; 85.0 min: 50% B2; 90.0 min: 100% B2. Modulation time of 2.0 min. Run time 100 min.

The chromatographic retention indexes of analytes are reported in Table S1 and were calculated as follow:

$$k = t_r – t_0/t_0 \qquad (S1)$$

Raw data registered through ChemStation® software were converted to a data matrix in GC image 2024 software (GCimage, Lincoln, U.S.A.) and then by Origin2024b (OriginLab Corporation,Northampton, U.S.A.) contour plots were created.

Table S1. Logarithms of chromatographic retention factors determined in bidimensional chromatography (log k ^{BonusRP} and log k $^{IAM.MG.DD2}$) using parallel gradient.

Substances	log k $^{Bonus\ RP}$	log k $^{IAM.MG.DD2}$
17α-hydroxyprogesterone	1.19	0.32
2,4,6-Trichlorophenol	1.30	0.44
2,4-Dichlorophenol	1.30	0.35
2-Amino-4-nitrophenol	0.60	0.15
2-Chlorophenol	1.19	0.38
2-Nitro-p-phenylenediamine	0.18	0.40
4-Amino2-nitrophenol	0.40	0.47
Aminopyrine	0.78	0.48
Antipyrine	0.48	0.12
Benzoic acid	0.65	0.14
Benzyl alchol	0.30	0.38
Caffeine	0.18	0.10
Chlorxylenol	1.29	0.30
Cortexolone	1.10	0.42
Cortexone	1.20	0.29
Corticosterone	1.10	0.42
Cortisone	1.10	0.42
Estriol	1.30	0.16
Ethyl nicotinate	0.90	0.41
Flurbiprofen	1.30	0.35
Haloperidol	1.18	0.39
Hydrocortisone	1.11	0.40
Ibuprofen	1.30	0.35
Indomethacin	1.30	0.35
Ketoprofen	1.29	0.47
Lidocaine	1.23	0.39
m-Cresol	0.88	0.43
Methyl -4-hydroxybenzoate	1.13	0.37
Methyl nicotinate	0.48	0.44

Naproxene	1.13	0.30
o-cresol	0.88	0.43
Paracetamol	0.40	0.13
p-Cresol	0.88	0.43
Phenol	0.81	0.44
p-Nitrophenol	1.23	0.20
Prednisolone	1.23	0.16
Progesterone	1.30	0.16
Resorcinol	1.31	0.26
Salicylic acid	1.00	0.13
Thymol	1.30	0.16
Triamcinolone	1.00	0.41
Triamcinolone acetonide	1.22	0.41
β-naphthol	1.26	0.31

.

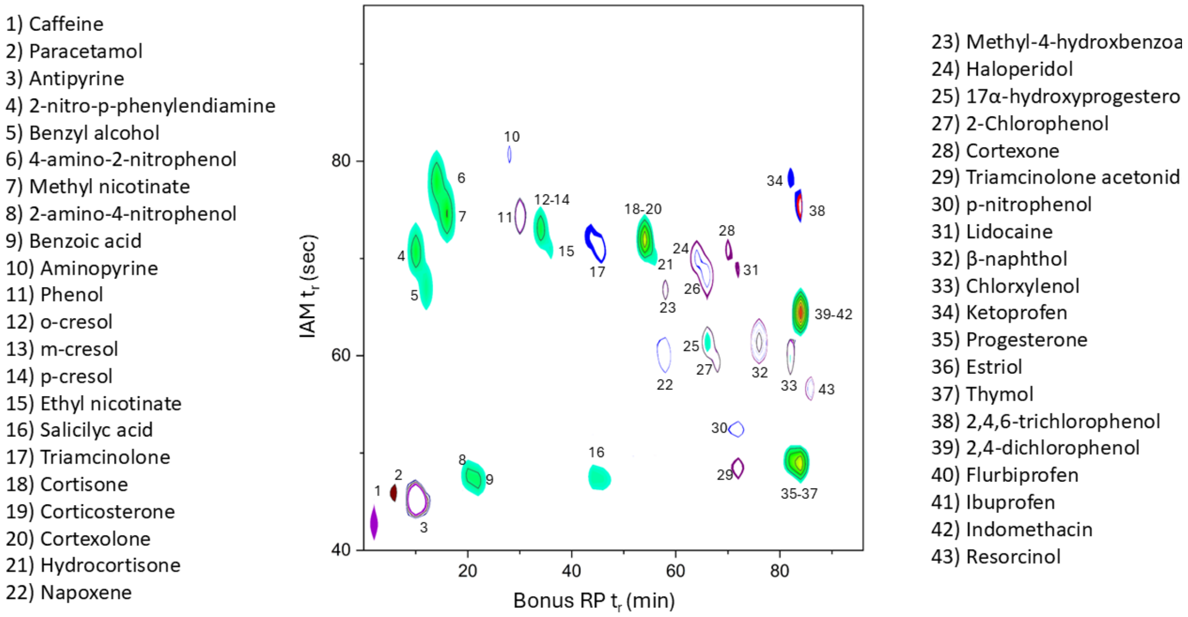

Figure S1. Contour plot recorded at λ 230 nm on Zorbax BONUS-RP × IAM.PC.DD2, using parallell gradient elution, of 43 chemicals in the dataset
